# Visualization of the photodegradation of a therapeutic drug by chemometric-assisted fluorescence spectroscopy†

**DOI:** 10.1039/d2ra03534k

**Published:** 2022-07-19

**Authors:** Masaru Tanioka, Tsugumi Ebihana, Manae Uraguchi, Haruka Shoji, Yuka Nakamura, Rina Ueda, Shota Ogura, Yoshifumi Wakiya, Tohru Obata, Takahiro Ida, Jun Horigome, Shinichiro Kamino

**Affiliations:** School of Pharmaceutical Sciences, Aichi Gakuin University 1-100 Kusumoto-cho, Chikusa-ku Nagoya 464-8650 Japan skamino@dpc.agu.ac.jp; Sony Group Corporation 1-7-1 Konan Minato-ku Tokyo 108-0075 Japan; Hitachi High-Tech Science Co., Ltd Hitachinaka-shi Ibaraki 312-0052 Japan

## Abstract

The fluorescence spectral fingerprint, also known as the excitation-emission matrix (EEM), is used to assess and visualize therapeutic drug photodegradation in combination with chemometrics. Examination of EEM-parallel factor analysis (PARAFAC) data showed that an individual component was easily separated from a mixture of photogenerated products of a heterocyclic pharmacophore, in this case, phenothiazine drugs (PTZs). Detailed investigations of both structure–EEM relationships and kinetics revealed that the components extracted from EEM–PARAFAC could be quantitatively attributed to such photogenerated products as phenothiazine sulfoxide and carbazole derivatives. EEM in combination with principal component analysis (PCA) could be used as a mapping tool to visualize information of the photodegradation process of PTZs. We also assessed the photostability of various types of PTZs containing side chains by using validated EEM–PARAFAC methodology.

## Abstract 2

Drug quality and assurance changes with time under the influence of a variety of environmental factors, such as light, temperature, and moisture.

## Graphical abstract

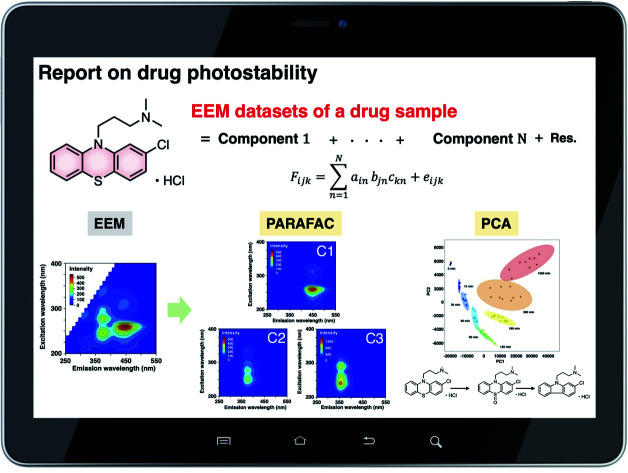

## 1. Introduction test

Drug quality and assurance changes with time under the influence of a variety of environmental factors, such as light, temperature, and moisture.^1–4^ Light is one of the most serious causes of the degradation of heterocycles in the active pharmacophore of a drug.^5–11^ This physical trigger not only reduces efficacy but also produces toxic effects brought about by the generation of photodegradation products.^12^ Thus, the assessment of drug photostability is essential to ensure both proper use and patient safety. Analytical techniques for the quality assessment of drugs include high-performance liquid chromatography (HPLC) and liquid chromatography tandem mass spectrometry (LC-MS). Although those methods have high precision, accuracy, and sensitivity, they involve high cost and complicated operations.^13,14^ A superior analytical technique is required to further advance both drug discovery and healthcare.

The fluorescence fingerprint, also known as the excitation-emission matrix (EEM), in combination with parallel factor analysis (PARAFAC), a multiway analysis, has recently attracted much interest.^15^ PARAFAC can decompose an EEM dataset into various chemical components. The application of EEM–PARAPAC has been explored in food quality evaluation and environmental monitoring.^16–21^ This approach has also been applied to the degradation analysis of pesticides and herbicides.^22,23^ Recently, Arques and co-workers employed the methodology to monitor the oxidation process of fluoroquinolone antibiotics;^24^ however, this approach is underutilized in the stability assessment of therapeutic drugs. Furthermore, detailed studies of structure–EEM relationships in the degradation process are necessary for users to understand the validity of the PARAFAC model. Herein, we report the use of EEM–PARAFAC in the assessment of therapeutic drug photostability; this approach was selected because of its simple and rapid operation and cost performance. In addition, the developed methodology enables the visualization of information of the photodegradation process.

## 2. Experimental section

### 2.1. Materials

Chlorpromazine hydrochloride (1), levomepromazine maleate (6), and prochlorperazine dimaleate (7) were purchased from Tokyo Chemical Industry Co., Ltd (Tokyo, Japan). Other reagents and solvents were purchased from Kanto Chemical Co., Inc. (Tokyo, Japan), Tokyo Chemical Industry Co., Ltd (Tokyo, Japan), and FUJIFILM Wako Pure Chemical Co. (Osaka, Japan). All solvents were used without further purification. Silica gel chromatography was conducted over TLC Silica gel 60 F254 (Merck). Developed TLC plates were visualized under a short-wave UV lamp and by heating plates that were dipped in ammonium phosphomolybdate sulfate solution.

### 2.2. Instruments

UV-vis spectra were recorded on a U-2900 spectrophotometer (HITACHI High-Technologies Co., Ltd, Tokyo, Japan). Absorption measurements over the spectral range of 250 nm to 450 nm were carried out in quartz cells having an optical path length of 1 cm. ^1^H NMR and ^13^C NMR spectra were recorded on a JEOL ECZ-400S spectrometer (JEOL Ltd, Tokyo, Japan). The solvent used for NMR measurements was CDCl_3_. ESI mass spectra were measured on an Agilent Technologies 6230 LC/TOF mass spectrometer (Agilent Technologies Japan, Ltd, Tokyo, Japan).

### 2.3. Photodegradation reaction

To oxygen-purged solvent (10 mL) in a dry glass tube with a two-way stopcock were added phenothiazine drugs (PTZs), and an LED lamp (PER-AMP, Techno Sigma Ltd, Okayama, Japan) was immersed into the reaction mixture. The reaction mixture was then irradiated with the LED lamp at 365 nm in the dark condition at 293 K. The measurement sample size was nine times (*n* = 9) in the experimental.

### 2.4. Excitation and emission matrixes

Excitation-emission matrixes (EEMs) of PTZs were measured on an F-7100 fluorescence spectrophotometer (HITACHI High-Technologies Co., Ltd, Tokyo, Japan) at excitation wavelengths ranging from 200 to 400 nm (1 nm intervals) and emission wavelengths ranging from 250 to 550 nm (5 nm intervals). Accurate measurement of EEM was carried out by using rhodamine B concentrated solution as the reference material. A cut filter was utilized to eliminate scattered light. All solvents for EEM measurement were purchased from Nacalai Tesque (Kyoto, Japan). After the photodegradation reaction reached completion, the solution was transferred into a quartz cell for EEM measurement without pretreatment.

### 2.5. Chemometric analyses

A PARAFAC model was used to separate components on the basis of the variation of excitation and emission wavelengths in the EEM dataset of a drug sample. The EEM dataset of a drug sample *k* is the sum of multiple components (*n* = 1…*N*) arranged in a matrix product of trilinear structures of excitation wavelength (*a*_*in*_), emission wavelength (*b*_*jn*_), and concentration (*c*_*kn*_). EEM is expressed as a set of trilinear terms and a residual matrix, as shown in eqn (1). *F*_*ijk*_ is the fluorescence intensity of drug sample *k* at excitation (*i*) and emission (*j*) wavelengths; *a*_*in*_ and *b*_*jn*_ are the excitation and emission wavelengths, respectively; *c*_*kn*_ is the concentration of component *n* in drug sample *k*; and *e*_*ijk*_ is the residual representing the unexplained variation.
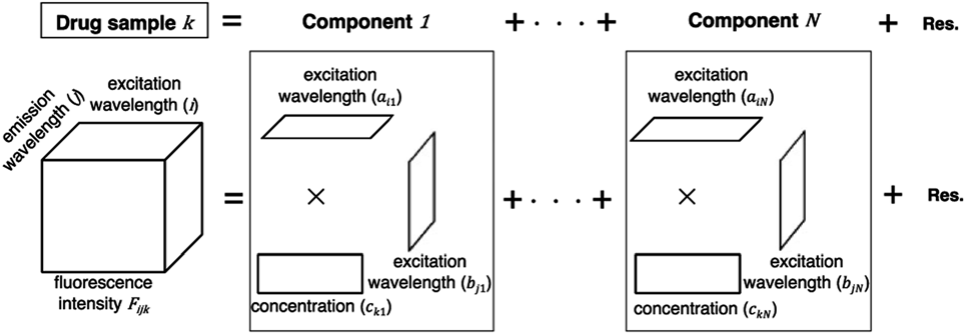
1
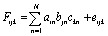


PCA was used to investigate the time-dependent photodegradation process of PTZs. To evaluate the measurement error, the sample was exchanged each time and measurement was repeated 9 times. The total percentage of explained variances of PC1 and PC2 was 98% or higher. Therefore, a PCA biplot showing the two principal components PC1 and PC2 was prepared. Chemometric analyses of PARAFAC and PCA data were carried out by using 3D SpectAlyze software (Dynacom Ltd, Chiba, Japan).

### 2.6. Trapping and characterization of photogenerated products

Compound 1: (35.5 mg, 10 mmol) was added to oxygen-purged methanol (10 mL) in a dry glass tube with a two-way stopcock. The reaction mixture was irradiated with an LED lamp at 365 nm in the dark condition at 293 K for 18 h. After the completion of the reaction, the solvent was evaporated *in vacuo*. The crude products were purified by silica gel preparative chromatography.

Compound 2: ^1^H NMR (400 MHz, CDCl_3_): *δ* = 7.91 (dd, *J* = 8.0, 1.6 Hz, 1H), 7.83 (d, *J* = 8.0 Hz, 1H), 7.67–7.62 (m, 1H), 7.56 (d, *J* = 1.6 Hz, 1H), 7.52 (d, *J* = 8.0 Hz, 1H), 7.29–7.28 (m, 1H), 7.22 (dd, *J* = 8.0, 1.6 Hz, 1H), 4.40–4.34 (m, 2H), 2.64–2.60 (m, 2H), 2.37 (s, 6H), 2.22–2.15 (m, 2H). ^13^C NMR (100 MHz, CDCl_3_): *δ* = 139.7, 139.2, 138.1, 133.3, 132.6, 131.5, 125.4, 123.6, 122.7, 122.4, 116.6, 56.0, 45.7, 45.0, 24.2. HRMS (ESI, positive mode): *m*/*z* [M]^+^ calculated for C_17_H_20_ClN_2_OS 335.0985; found 335.0985.

Compound 3: ^1^H NMR (400 MHz, CDCl_3_): *δ* = 8.06–8.04 (m, 1H), 7.98 (d, *J* = 8.4 Hz, 1H), 7.49 (d, *J* = 2.0, 1H), 7.47–7.46 (m, 2H), 7.26–7.22 (m, 1H), 7.18 (dd, *J* = 8.4, 2.0 Hz, 1H), 4.38–4.35 (m, 2H), 2.29–2.23 (m, 8H), 2.05–1.98 (m, 2H). ^13^C NMR (100 MHz, CDCl_3_): *δ* = 141.3, 140.9, 131.5, 126.1, 122.5, 121.5, 121.2, 120.4, 119.5, 119.4, 109.2, 109.1, 56.4, 45.5, 40.7, 26.9. HRMS (ESI, positive mode): *m*/*z* [M]^+^ calculated for C_17_H_20_ClN_2_: 287.1315; found 287.1310.

Compound 4: HRMS (ESI, positive mode): *m*/*z* [M + H]^+^ calculated for C_17_H_19_ClN_2_OS: 355.0985; found 355.0978.

Compound 5: ^1^H NMR (400 MHz, CDCl_3_): *δ* = 7.84 (dd, *J* = 8.0, 1.6 Hz, 1H), 7.55–7.51 (m, 1H), 7.28–7.23 (m, 3H), 7.17–7.13 (m, 1H), 7.15–7.12 (m, 1H), 3.90 (t, *J* = 6.8 Hz, 2H), 2.37 (t, *J* = 6.8 Hz, 2H), 2.19 (s, 6H), 1.80–1.77 (m, 2H). ^13^C NMR (100 MHz, CDCl_3_): *δ* = 144.4, 143.1, 141.3, 134.2, 132.9, 130.1, 129.3, 126.0, 124.3, 124.1, 123.0, 121.2, 56.6, 50.1, 45.6, 25.8. HRMS (ESI, positive mode): *m*/*z* [M + H]^+^ calculated for C_17_H_19_ClN_2_O_3_S: 367.0883; found 367.0879.

### 2.7. Crystallographic data collection and structure refinement

Single crystals of compounds 1 and 2 were obtained by slow diffusion of less solubilizing solvent vapor (Et_2_O) into more solubilizing solvent (CHCl_3_) at 10 °C. Crystal data for these crystals are summarized in Table S1.† X-ray diffraction data were collected on a Rigaku XtaLAB Synergy-i diffractometer using CuKα radiation at 103.15 K.

### 2.8. Computational details

All calculations were performed at the density functional theory (DFT), by means of B3LYP functional level as implemented in Gaussian 09.^25^ The 6-31G(d,p) basis set was used for all atoms. Excitation wavelengths and oscillator strengths were obtained at the density functional level using time-dependent perturbation theory (TDDFT) approach. Vibrational frequency computations verified the nature of the stationary points.

### 2.9. Kinetics

Kinetic studies were carried out in CH_3_OH solution at 298 K. A 10 μM solution of 1 was prepared in oxygen-purged CH_3_OH, and EEMs were measured before and after UV irradiation. The following reaction shows the photodegradation process of 1. In the reaction, *k*_1_ is the rate constants for the first step.
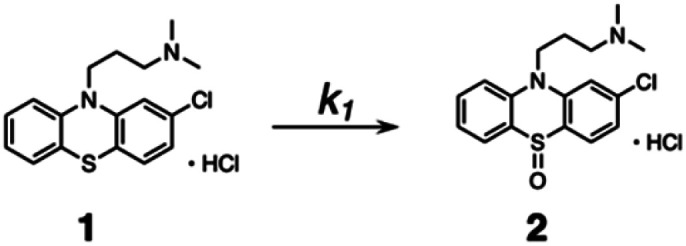


The differential equations for this reaction are expressed as follows:2
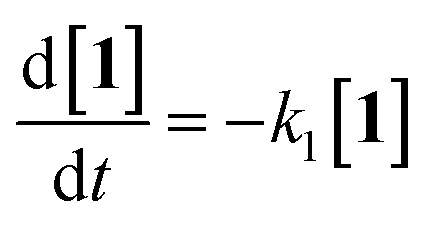


These differential forms can be transformed and integrated to derive equations for three time-dependent concentrations. [1]_0_ is the initial concentration of 1 and *t* is the UV irradiation time.

3 [**1**] = [**1**]_0_e^−*k*_1_*t*^

Eqn (2) was used to calculate the relative excitation and emission wavelength loading scores (*Q*_em_) of component, C1, where *I*_*t*_ is the loading score and *I*_0_ is the loading score at *t* = 0 (Fig. S1†). The concentration of 1 was determined by multiplying the initial concentration of 1 by *Q*_em_. The pseudo-first-order constant *k*_1_ was calculated using eqn (3) (Fig. S2†).4
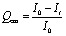


The value of *k*_1_ was also fitted to eqn (2) by using the Levenberg–Marquardt method. A nonlinear curve fitting program was prepared, and the source code of Python was disclosed in ESI.†

## 3. Result and discussion

**Fig. 1:**
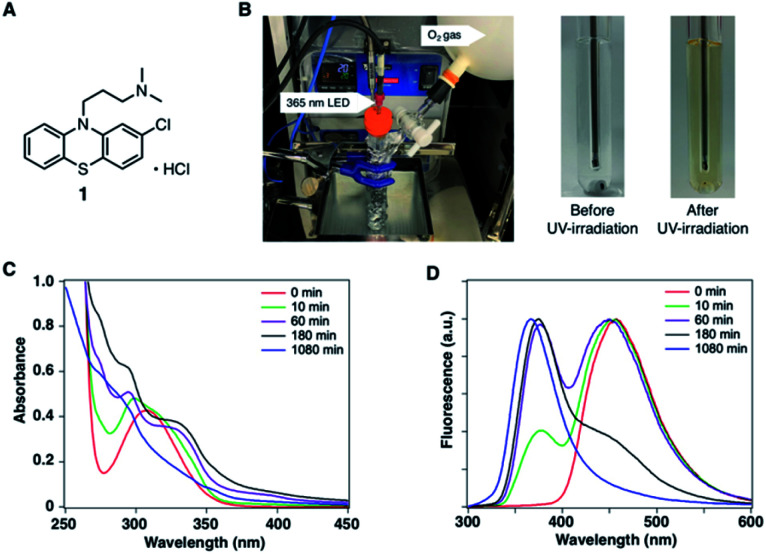
(A) Chemical structure of chlorpromazine hydrochloride (1). (B) Photodegradation reaction of 1 in CH_3_OH. Photographs show the photodegradation of 1 before and after UV irradiation. (C) Absorption and (D) fluorescence spectra of 10 μM of 1 in CH_3_OH before and after UV irradiation.

In the development of analytical methodology for drug quality assessment based on EEM–PARAFAC, we selected phenothiazine drugs (PTZs), which are typically used to treat psychosis and palliate end-of-life symptoms, because PTZs are susceptible to light-induced physicochemical changes.^26^Fig. 1 shows the absorption and fluorescence spectra of chlorpromazine hydrochloride (1) in oxygen-purged methanol upon UV irradiation at 365 nm. All measurements were performed at 293 K. Before the UV irradiation, two main absorption bands in the UV region were observed for 1. The absorbance band observed around 255 nm is attributable to the π–π* transition. A weak band appearing in the longer wavelength region is attributed to the n–π* transition derived from sulfur lone-electron pairs (Fig. S3 and S4†). 1 showed blue fluorescence at approximately 450 nm. Upon UV irradiation, the absorption bands in the UV region were altered, and an absorption tail that extended into the long-wavelength region appeared. In addition, the solution of 1 changed from colourless to orange, which was visible to the naked eye. More importantly, the UV irradiation caused a blue shift of the fluorescence band, and a new fluorescence band appeared at approximately 370 nm. There was almost no difference in the absorption and fluorescence spectral behaviors among the solvents (Fig. S5†).

**Fig. 2:**
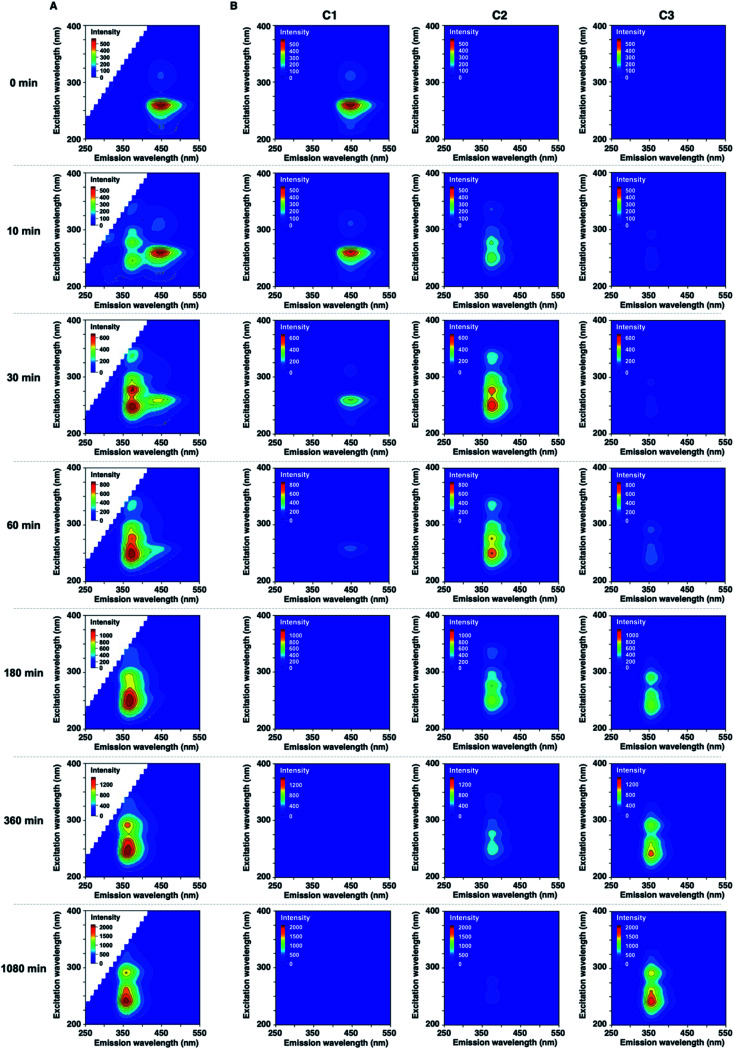
(A) Excitation-emission matrixes (EEMs) of 10 μM of 1 in CH_3_OH before and after UV irradiation. (B) Three-component fingerprints from EEM–PARAFAC.

We next measured the EEMs of 1 in methanol, as shown in Fig. 2A. Because concentration and fluorescence intensity showed a linear correlation from 5 to 25 μM of 1 (Fig. S6†), we conducted measurement using a 10 μM solution of 1. Before UV irradiation, the maximum emission wavelength was clearly observed at approximately 450 nm, which corresponded to the 260 and 310 nm excitation wavelengths derived from 1. Increasing the irradiation time resulted in a decrease in the intensities of 450/260 and 450/310 emission/excitation pairs. A new emission area spanning 300 nm to 450 nm for the 200 to 350 nm excitation emerged with the further photodegradation of 1.

Statistical EEM–PARAFAC was performed to separate the individual components from the photodegraded matrix of 1. EEM–PARAFAC revealed three independent components. The appropriateness of the number of components in the PARAFAC model was then evaluated from the core consistency diagnostic (Fig. S7†). Higher than 91% core consistencies were obtained for the three components. The three components, C1, C2, and C3, are described in Fig. 2B. C1 is represented by 450/260 and 450/310 emission/excitation pairs. The intense C1 is attributable to compound 1. A blueshift of the emission was observed for C2 and C3 relative to C1. The emission/excitation pairs of 375/250, 375/275, and 375/335 nm were confirmed for C2. It is clear that C3 has 355/240 and 355/290 nm emission/excitation pairs.

**Fig. 3:**
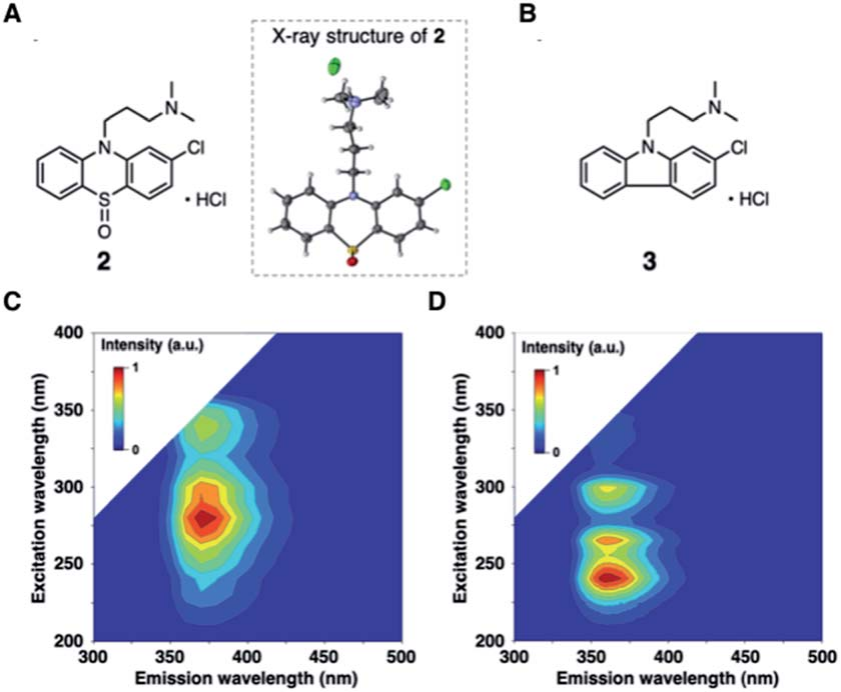
(A) Chemical and X-ray crystal structures of phenothiazine hydrochloride sulfoxide (2). ORTEP view of 2 with thermal ellipsoids shown at 50% probability level. (B) Chemical structure of 2-chloro-*N*,*N*-dimethylcarbazole (3). EEMs of isolated photodegradation products of (C) 2 and (D) 3 in CH_3_OH.

To further validate the EEM–PARAFAC methodology for the quality assessment of drugs, we attempted to trap and characterize photogenerated products from the reaction of 1. The reaction was performed with 10 mM of 1 in oxygen-purged methanol for 18 h under UV irradiation, and the reaction mixture was then separated by silica gel chromatography. As expected, compound 1 disappeared, and phenothiazine hydrochloride sulfoxide (2) was obtained as the major product in 55% yield. The crystal structure of 2 was successfully established by single-crystal X-ray diffraction analysis (Fig. 3A). The alkylamine substituent of 2 was identified to be a hydrochloride. The phenothiazine ring of 2 had higher planarity than that of 1 (Fig. S8† shows the crystal structure of 1). From ^1^H and ^13^C NMR spectral measurements, we isolated 2-chloro-*N*,*N*-dimethylcarbazole (3). Small amounts of the oxidation product of C_17_H_19_ClN_2_SO (4) and a further oxidation product of C_17_H_19_ClN_2_SO_3_ (5) were trapped, respectively. In addition, multiple coloured compounds were obtained, as confirmed by silica gel thin-layer chromatography.

The EEMs of the isolated photodegradation products of 1 were measured to assign C2 and C3 extracted from PARAFAC. The EEMs of compounds 2 and 3 are shown in Fig. 3 (Fig. S9† shows the EEMs of 4 and 5). The emission/excitation pairs of C2 matched the 375/275 and 375/335 nm emission/excitation pairs of 2. The emission/excitation pairs of C3 matched the 355/240, 355/∼265, and 355/290 nm emission/excitation pairs of 3.

**Fig. 4:**
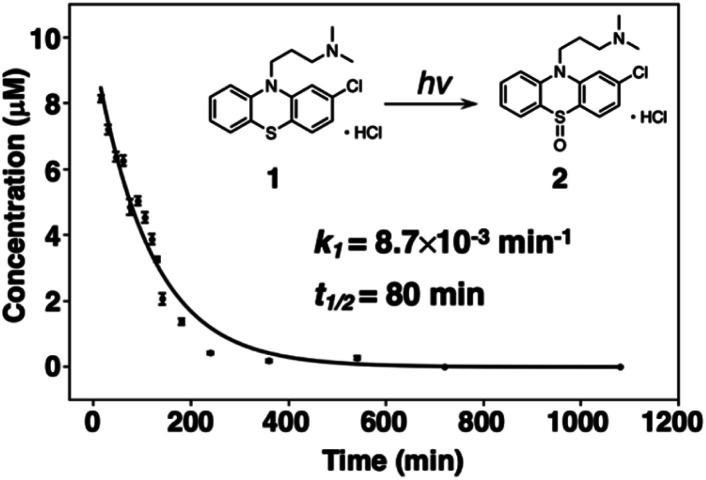
Time-dependent concentration profile for the photodegradation of 1 at 293 K. Inset shows the pseudo-first-order constant *k*_1_ obtained from a nonlinear curve fitting program.

The decomposition of three-way arrays has motivated us to further explore quantitative information on the photodegradation process of PTZs. Fig. 4 shows the time-dependent concentration profiles of 1 from EEM–PARAFAC. The profiles reveal that the photodegradation of 1 was fitted to the nonlinear equation of pseudo-first order kinetics. The pseudo-first-order constant *k*_1_ was calculated to be 7.3 × 10^−3^ min^−1^ at 293 K (Fig. S2†). We also used the nonlinear approximation by the Levenberg–Marquardt method. As a result, the value of *k*_1_ was fitted to be 8.7 × 10^−3^ min^−1^ with a half-life (*t*_1/2_) on the order of 79 min. From these results, it is clear that 1 is decomposed with a half-life on the order of 79 minutes after UV irradiation to give 2, and then 3 is formed from 2*via* a photosensitized reaction of 1 consistent with reference to the literature.^27^

**Fig. 5:**
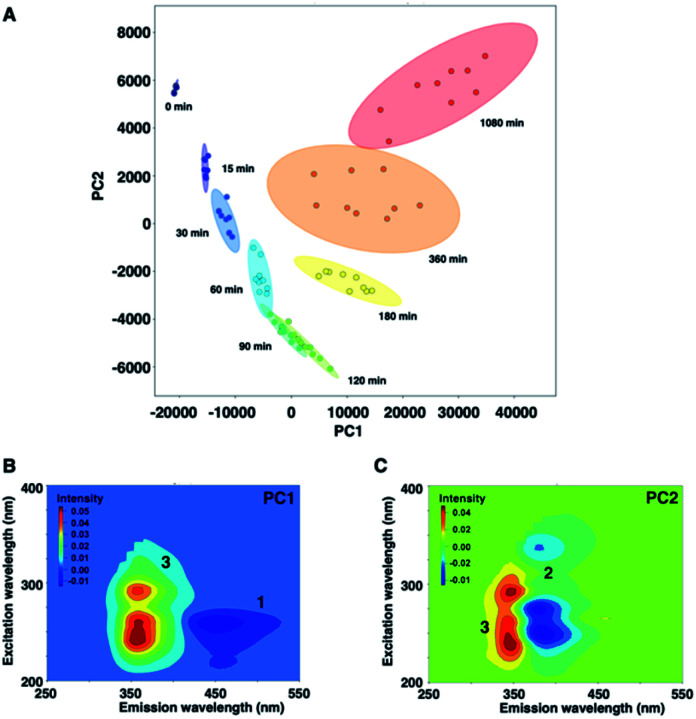
(A) PCA score plot of 1. Loading plots of (B) PC1 and (C) PC2.

Fig. 5 shows a PCA biplot that visualizes information on the time-dependent photodegradation of 1. The percentages of explained variances for PC1 and PC2 are 93.1 and 5.74%, respectively. The PC1 scores increased with irradiation time. In contrast, the PC2 scores decreased up to 60 min of UV irradiation, and increased thereafter. In the loading plots of PC1 and PC2, the EEMs showed a negative blue colour for 1 and 2, and a positive red colour for 3. For the photodegradation process of 1, PC1 is associated with the production of the carbazole form, and PC2 is related negatively to the oxidation process. From PCA, the time-dependent decrease of 1 was observed, and 2 was converted into 3 as UV irradiation time was increased to 1080 min.

**Fig. 6:**
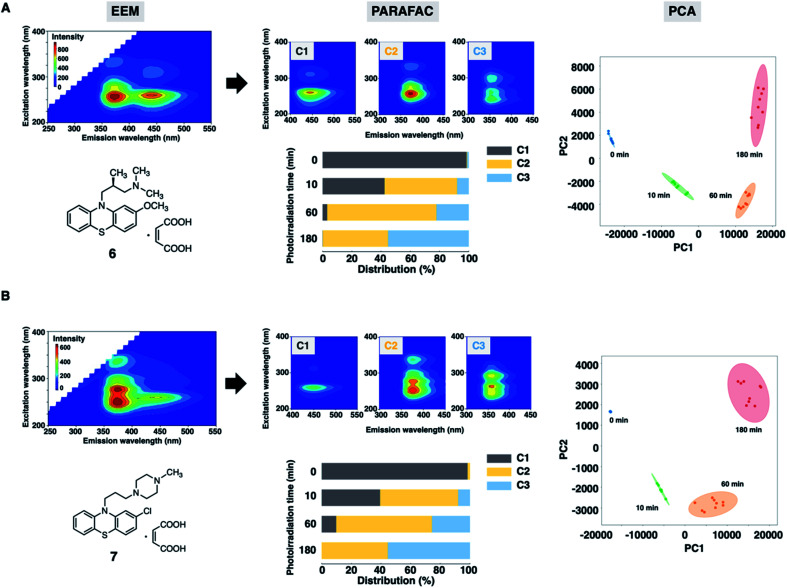
(Left) EEM and chemical structure of 10 μM of PTZs in CH_3_OH after UV irradiation for 10 min. (Middle) Three-component fingerprints and relative distribution calculated from EEM–PARAFAC. (Right) PCA score plots. (A) Levomepromazine maleate (6) and (B) prochlorperazine dimaleate (7).

Finally, we assessed the photodegradation of PTZs (6) and (7) containing an alkylamino and a piperazine side chain, respectively, by EEM–PARAFAC. The photostability results of 6 and 7 are shown in Fig. 6, S11 and S12.† Photodegradation occurred immediately to give the sulfoxide forms (C2) of 6 and 7. The relative distribution of the three components demonstrated that more than 50% of 6 and 7 (C1) was decomposed after UV irradiation for 10 min. The carbazole forms (C3) were produced later than C2 to show the distribution of approximately 55% after UV irradiation for 180 min. The PCA biplot of PC1 *vs.* PC2, which shows a parabolic curve, revealed that there was no significant difference in photostability due to the difference in substituents of PTZs. From our results, a simple and rapid analytical technique with the combination of fluorescence fingerprint and data science was established for the stability assessment of PTZs. Qualitative and quantitative information on the photodegradation process of PTZs was obtained without the separation of components by chromatographic column. This analytical batch technique also led to more cost-effectiveness, such as drug sample and solvent saving, than HPLC and LC-MS methods. It was widely applicable to the photostability assessment of various types of PTZs.

## 4. Conclusions

In summary, we have presented a simple and rapid method for the assessment of drug photodegradation by using fluorescence spectral fingerprint, also known as EEM, in combination with data science approaches. The novelty of this research is that EEM–PARAFAC enables easy separation of the desired component from a mixture of photodegradation products of PTZs, which are used to treat psychosis and palliate end-of-life symptoms. We have examined the structure–EEM relationships to validate this approach and understand the photodegradation process of PTZs. In addition, we have shown that PCA is a powerful mapping tool to visualize information on the photodegradation process of PTZs. We postulate that data science assisted fluorescence spectral fingerprint would open new doors for applications to drug discovery as well as the quality assessment of various fluorescent drugs.

## Conflicts of interest

There are no conflicts to declare.

## Supplementary Material

RA-012-D2RA03534K-s001
