# Supplementary material for: Visualization of the photodegradation of a therapeutic drug by chemometric-assisted fluorescence spectroscopy
Source: RSC Adv. 2022 Jul 19;12(32):20714–20. doi: 10.1039/d2ra03534k (PMC9295133; doi:10.1039/d2ra03534k)
Supplement: RA-012-D2RA03534K-s001 [file RA-012-D2RA03534K-s001.pdf]

Electronic Supplementary Material (ESI) for RSC Advances.
This journal is © The Royal Society of Chemistry 2022

**Electronic Supplementary Information for**

# Visualization of the photodegradation of a therapeutic drug by chemometric-assisted fluorescence spectroscopy

Masaru Tanioka,[a] Tsugumi Ebihana,[a] Manae Uraguchi,[a] Haruka Shoji,[a] Yuka Nakamura,[a] Rina Ueda,[a] Shota Ogura,[a] Yoshifumi Wakiya,[a] Tohru Obata,[a] Takahiro Ida,[b] Jun Horigome,[c] Shinichiro Kamino*[a]

[a] School of Pharmaceutical Sciences, Aichi Gakuin University, 1-100 Kusumoto-cho, Chikusa-ku, Nagoya 464-8650, Japan

[b] Sony Group Corporation, 1-7-1 Konan Minato-ku, Tokyo 108-0075, Japan

[c] Hitachi High-Tech Science Co., Ltd., Hitachinaka-shi, Ibaraki 312-8504, Japan

## Table of Contents

**1. Figures**

**Fig. S1.** Fluorescence intensity scores of each components by the PARAFAC model.

**Fig. S2.** Decay profiles for first-order reactions of photodegradation process of **1**.

**Fig. S3.** Optimized structures and calculated absorption spectra of MCPZ.

**Fig. S4.** Frontier molecular orbitals and calculated absorption wavelength of MCPZ.

**Fig. S5.** Absorption spectra of 10 μM of **1** in (a) $CH_2Cl_2$, (b) $CH_3CN$, (c) DMF, and (d) $H_2O$ before and after UV irradiation.

**Fig. S6.** Calibration curve of the changes in fluorescence emission intensity at 450 nm of **1**.

**Fig. S7.** Core consistency diagnostic calculated for **1** individual PARAFAC model.

**Fig. S8.** Crystal structures of (a) **1** and (b) **2**.

**Fig. S9.** EEMs of isolated photodegradation products of (a) **4** and (b) **5** in $CH_3OH$.

**Fig. S10.** (a) Normalized fluorescence emission intensity, and (b) relative distribution calculated from EEM-PARAFAC of **1** in $CH_3OH$.

**Fig. S11.** (a) EEMs of 10 μM of **6** in $CH_3OH$ before and after UV irradiation. (b) Three-component fingerprints from EEM-PARAFAC.

**Fig. S12.** (a) EEMs of 10 μM of **7** in $CH_3OH$ before and after UV irradiation. (b) Three-component fingerprints from EEM-PARAFAC.

**2. Crystallographic data collection and structure refinement**

**3. NMR spectral data**

**4. Cartesian Coordinates (in Å) and Energies**

## 1. Figures

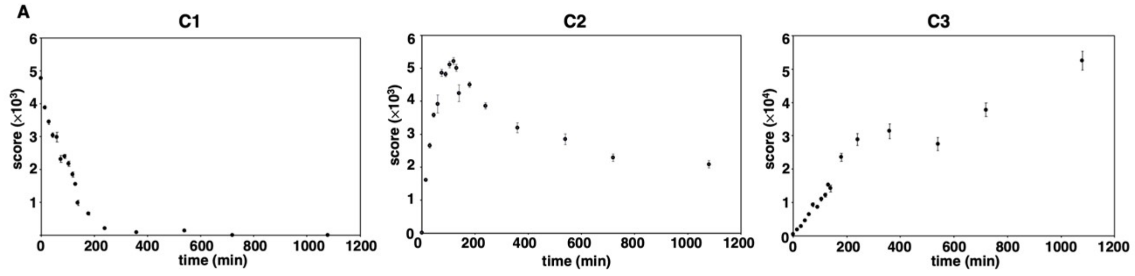


**Fig. S1.** Fluorescence intensity scores of each components for **1** in $CH_3OH$ by the PARAFAC model.

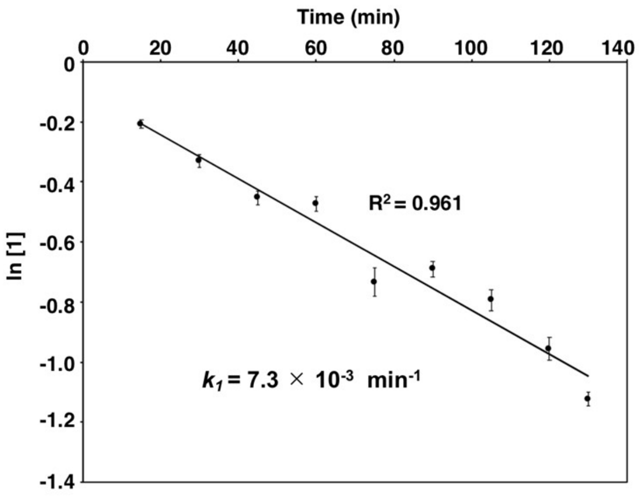


**Fig. S2.** The ln [**1**] vs. time plots for the photodegradation process of **1**. The curves were fitted with first-order kinetics to calculate the thermal bleaching rate constant: $k_{293\ K}$ = 7.3 × $10^{-3}$ $min^{-1}$.

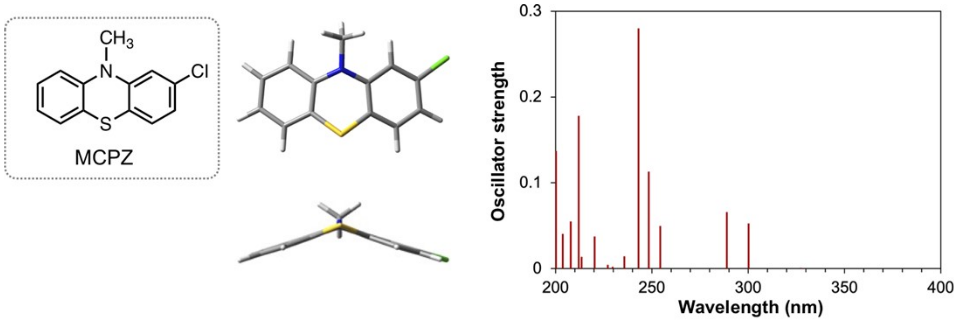


**Fig. S3.** Optimized structures and calculated absorption spectra of MCPZ. We carried out the density functional theory (DFT) calculations at the B3LYP/6-31G(d,p) level. The excitation energies and oscillator strengths for the optimized structures of MCPZ were obtained using the time-dependent DFT method.

a)

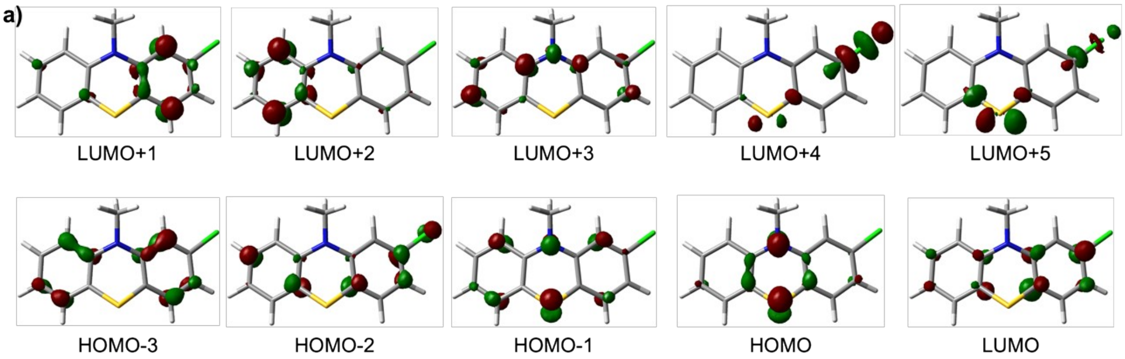


b)

| Excited state | Wavelength [nm] | *f* | Contribution |
|---|---|---|---|
| 1 | 327.36 | 0.0010 | HOMO → LUMO (95%)<br>HOMO → LUMO+1 (2%) |
| 2 | 300.17 | 0.0527 | HOMO → LUMO+1 (88%)<br>HOMO–1 → LUMO (3%)<br>HOMO → LUMO+2 (2%) |
| 3 | 288.90 | 0.0659 | HOMO → LUMO+2 (87%)<br>HOMO–1 → LUMO (5%)<br>HOMO → LUMO+1 (3%) |
| 4 | 254.40 | 0.0496 | HOMO → LUMO+3 (88%)<br>HOMO–2 → LUMO (3%)<br>HOMO–3 → LUMO (2%) |
| 5 | 248.42 | 0.1131 | HOMO → LUMO+4 (60%)<br>HOMO–1 → LUMO (20%)<br>HOMO → LUMO+5 (12%) |
| 6 | 243.07 | 0.2800 | HOMO–1 → LUMO (59%)<br>HOMO → LUMO+4 (24%)<br>HOMO–2 → LUMO+1 (6%)<br>HOMO → LUMO+2 (4%) |
| 7 | 235.68 | 0.0142 | HOMO–1 → LUMO+1 (89%)<br>HOMO–2 → LUMO+2 (3%) |

**Fig. S4.** (a) Frontier molecular orbitals of MCPZ (isovalue = 0.08). (b) Calculated absorption wavelength (nm), oscillator strength (*f*), and major contribution (%) for MCPZ.

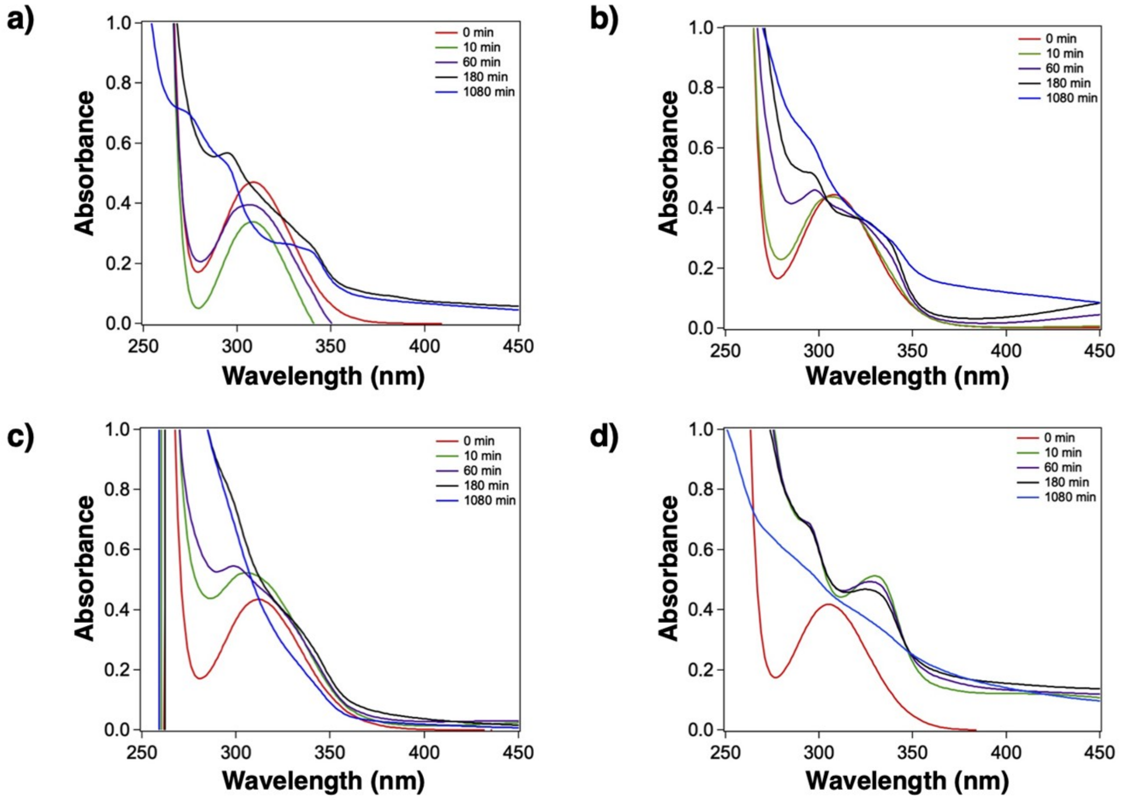


**Fig. S5.** Absorption spectra of 10 μM of **1** in (a) $CH_2Cl_2$, (b) $CH_3CN$, (c) DMF, and (d) $H_2O$ before and after UV irradiation.

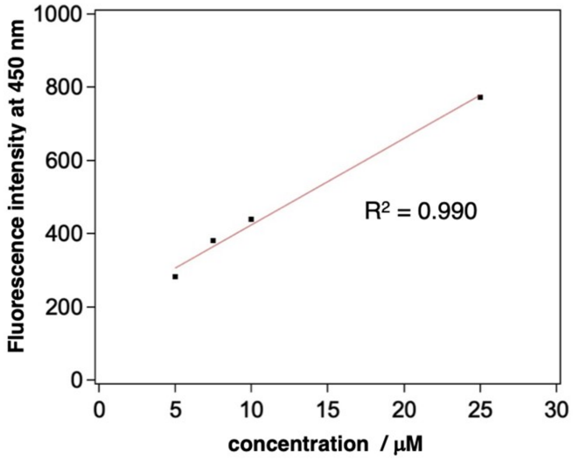


**Fig. S6.** Calibration curve of the changes in fluorescence emission intensity at 450 nm of **1**. All measurements were performed at 260 nm of excitation wavelength.

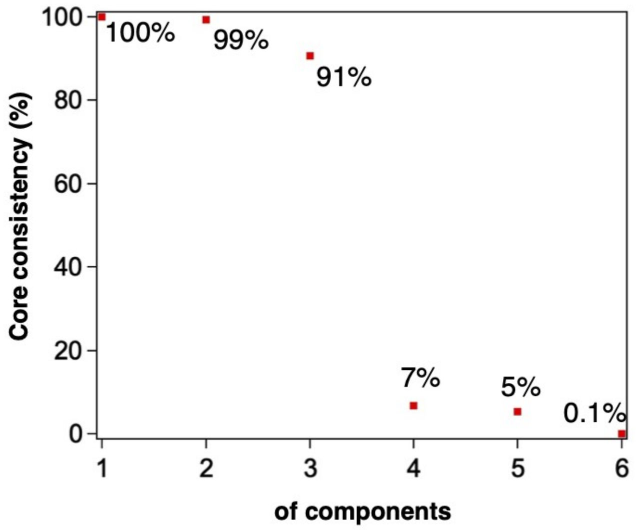


**Fig. S7.** Core consistency diagnostic calculated for **1** individual PARAFAC model.

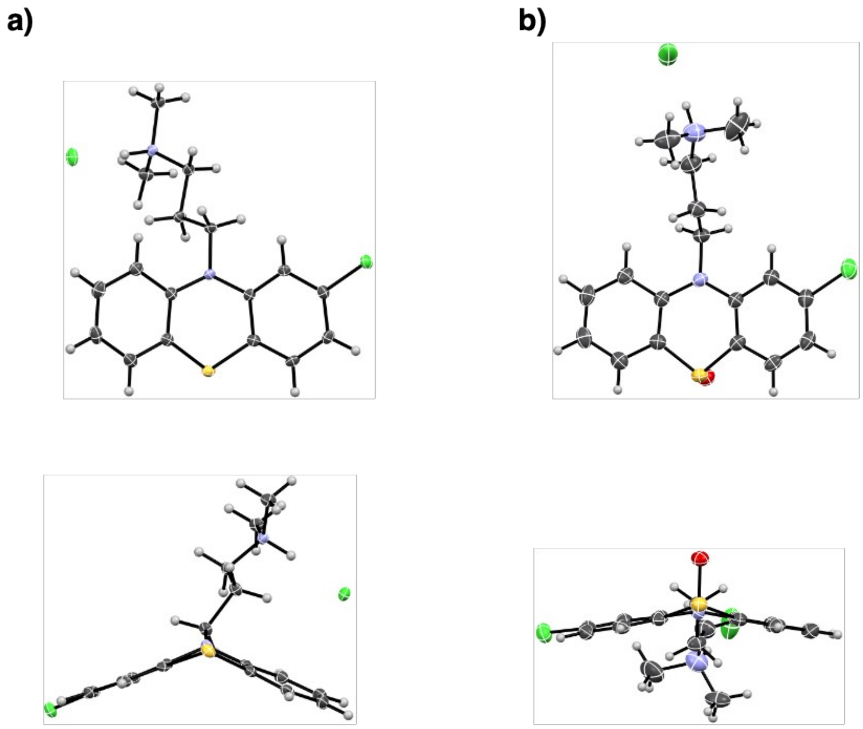


**Fig. S8.** Crystal structures of (a) **1** and (b) **2**.

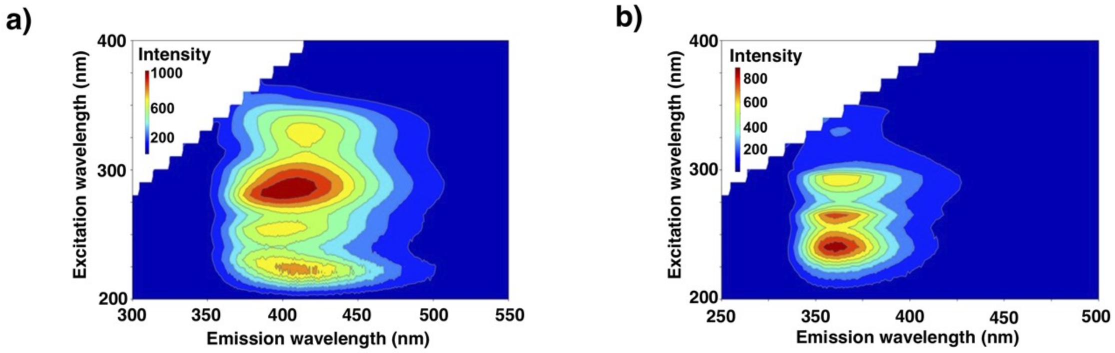


**Fig. S9.** EEMs of isolated photodegradation products of (a) **4** and (b) **5** in $CH_3OH$.

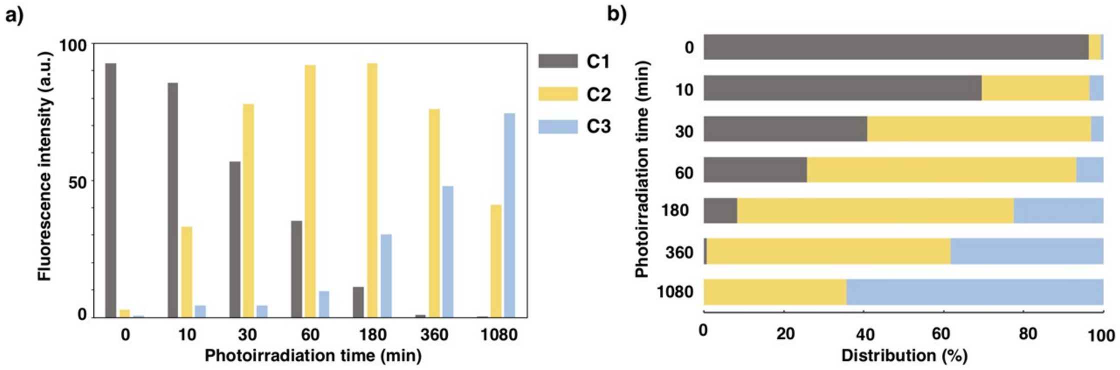


**Fig. S10.** (a) Normalized fluorescence emission intensity, and (b) relative distribution calculated from EEM-PARAFAC of **1** in $CH_3OH$.

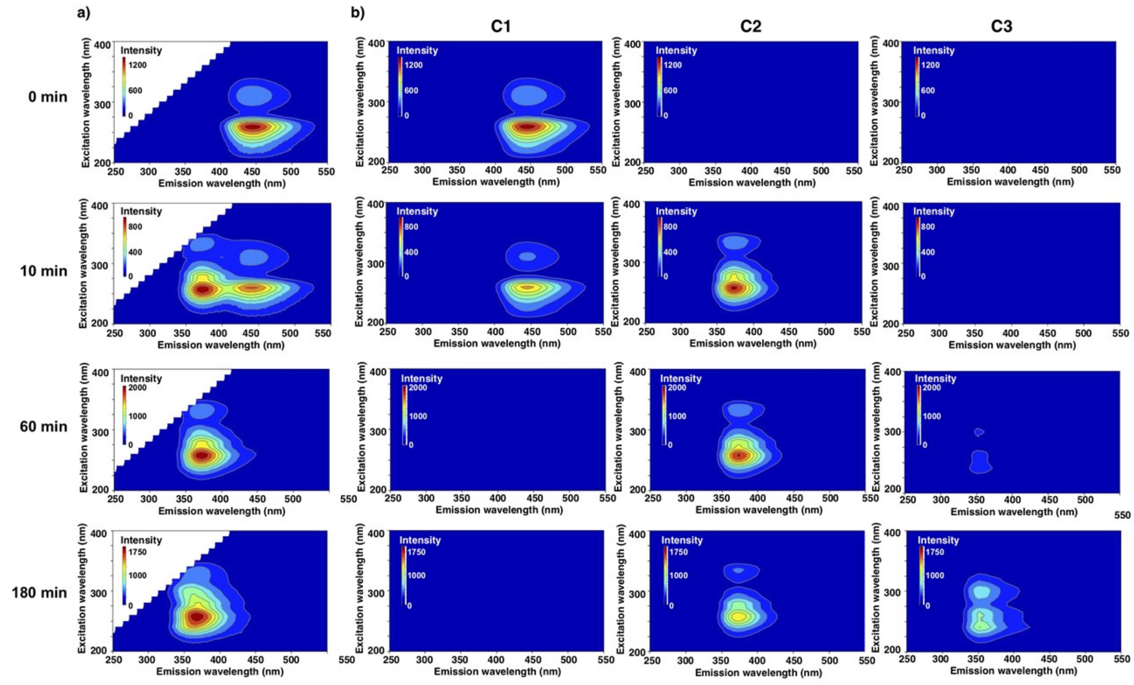


**Fig. S11.** (a) EEMs of 10 μM of **6** in $CH_3OH$ before and after UV irradiation. (b) Three-component fingerprints from EEM-PARAFAC.

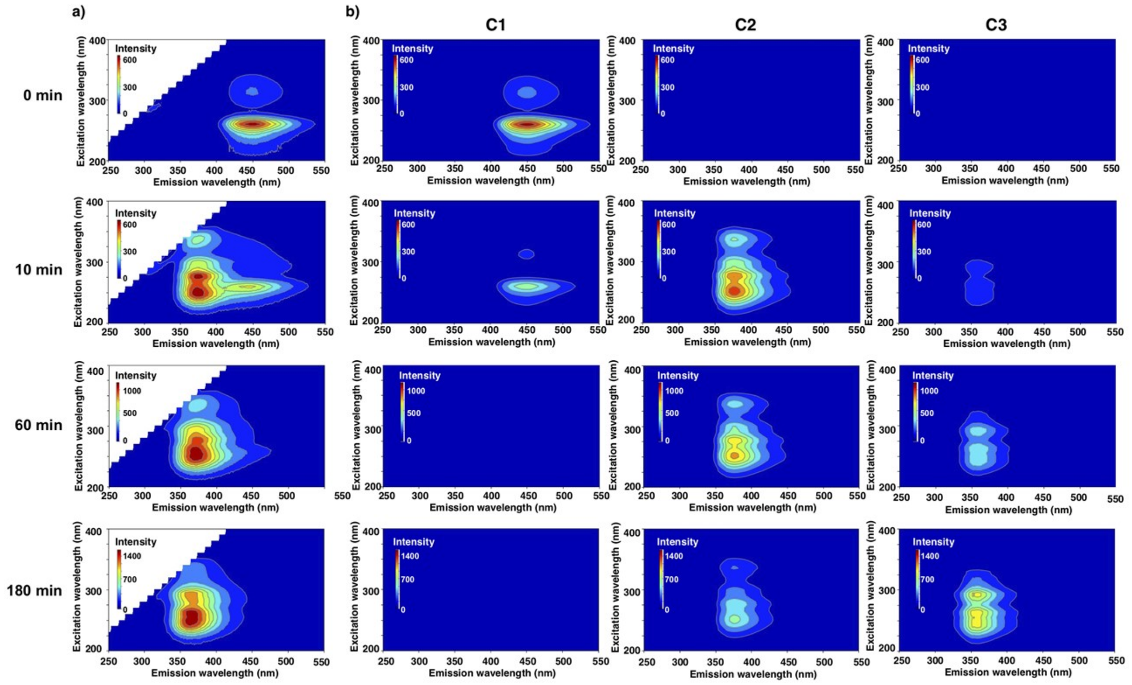


**Fig. S12.** (a) EEMs of 10 μM of **7** in $CH_3OH$ before and after UV irradiation. (b) Three-component fingerprints from EEM-PARAFAC.

## 2. Crystallographic data collection and structure refinement

**Table S1** Crystal data and structure refinement for compounds **1** and **2**.

| | **1** | **2** |
|---|---|---|
| Chemical formula | $2(C_{17}H_{20}Cl_2N_2S) \cdot H_2O$ | $C_{17}H_{20}Cl_2N_2OS$ |
| Formula Weight | 728.63 | 371.31 |
| Crystal Color, Habit | colorless, block | colorless, block |
| Crystal Dimensions | 0.391×0.263×0.119 mm | 0.118×0.074×0.032 mm |
| crystal system | monoclinic | triclinic |
| space group [No.] | $P\ 2_1/c$ | P 1 |
| $a$, Å | 11.88268(13) | 7.5231(2) |
| $b$, Å | 31.7078(4) | 8.4336(3) |
| $c$, Å | 9.56006(10) | 15.3632(5) |
| $\alpha$, ° | 90 | 75.928(3) |
| $\beta$, ° | 99.2494(11) | 89.441(3) |
| $\gamma$, ° | 90 | 84.786(2) |
| volume, $Å^3$ | 3555.15(7) | 941.51(5) |
| $Z$ | 4 | 2 |
| $D_{calcd}$, $g/cm^3$ | 1.361 | 1.310 |
| $T$, K | 103.15 | 103.15 |
| radiation[a] | CuKα | CuKα |
| $\mu$, $mm^{-1}$ | 4.386 | 4.172 |
| $2\theta_{max}$, ° | 68.428 | 68.186 |
| $F$(000) | 1528 | 388 |
| unique reflns | 6466 | 3393 |
| No. of parameters | 417 | 210 |
| R1 factor ($I > 2.00\sigma(I)$) | 0.0356 | 0.0695 |
| R factor (all reflection) | 0.0381 | 0.0751 |
| $wR$ factor | 0.0931 | 0.1845 |
| GOF | 0.946 | 1.055 |
| CCDC No. | 2101545 | 2101549 |

## 3. NMR spectra data.

Compound **2**

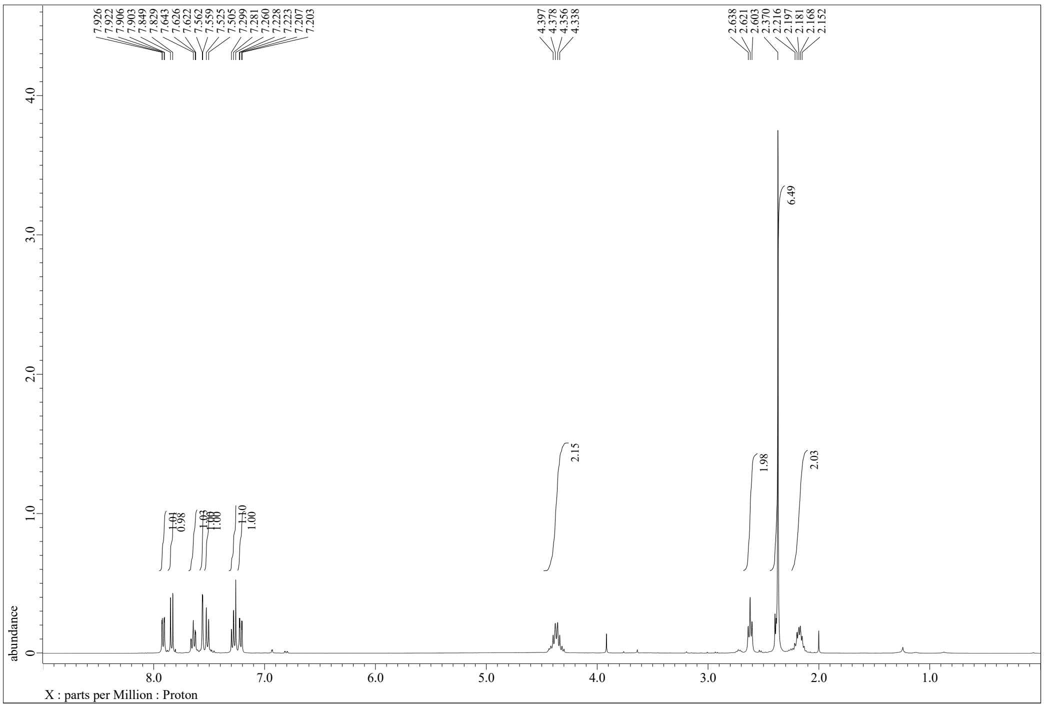


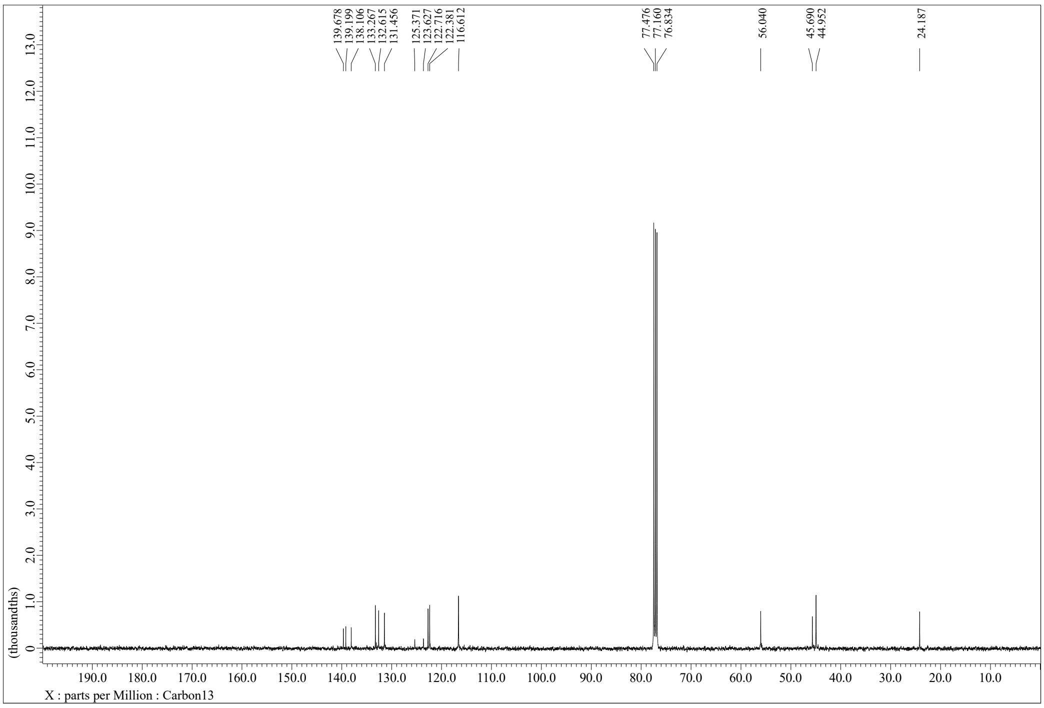

Compound **3**

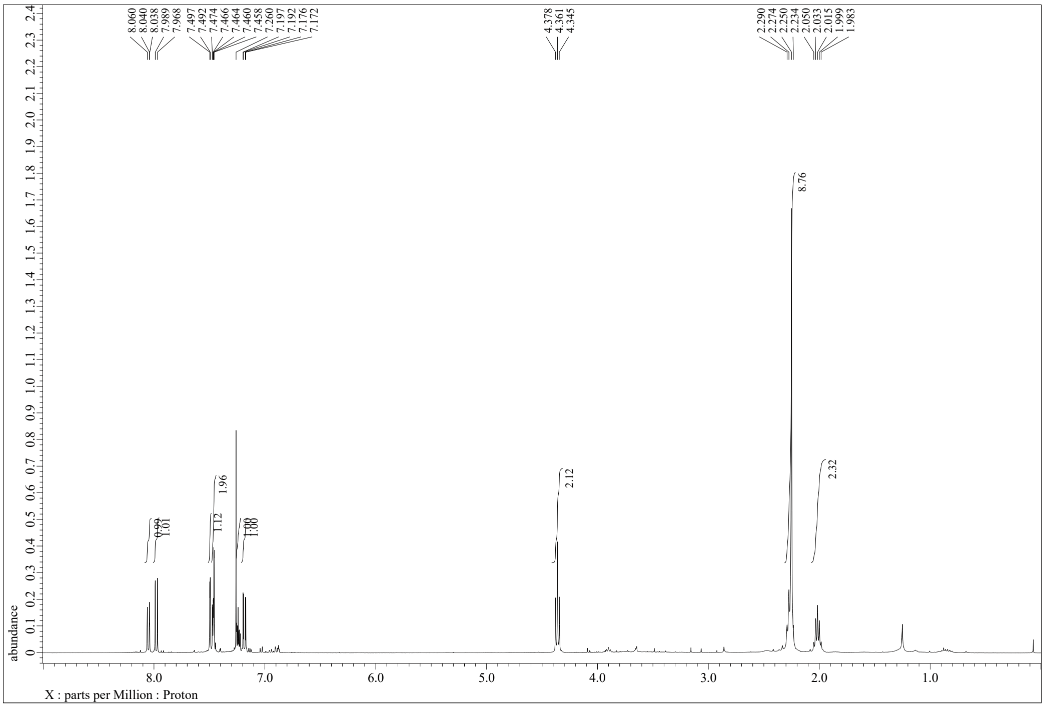

8.060
8.040
8.038
7.989
7.968
7.497
7.492
7.474
7.466
7.464
7.460
7.458
7.260
7.197
7.192
7.176
7.172
4.378
4.361
4.345
2.290
2.274
2.250
2.234
2.050
2.033
2.015
1.999
1.983
1.96
1.12
2.12
8.76
2.32
1.01
abundance
X : parts per Million : Proton


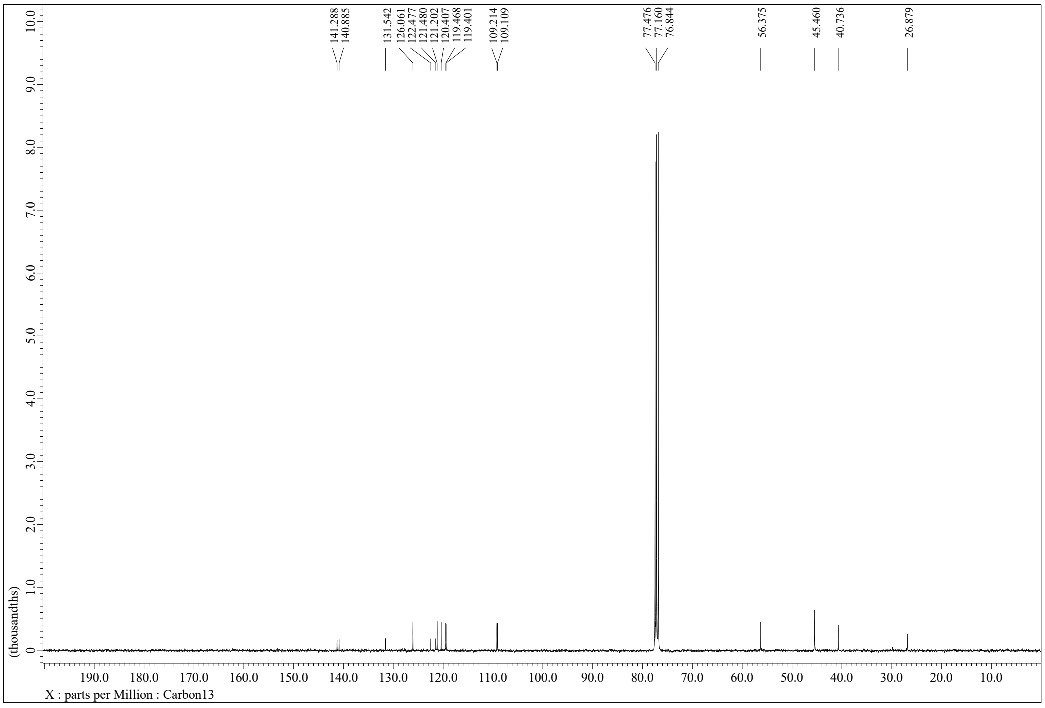

141.288
140.885
131.542
126.061
122.477
121.480
121.202
120.407
119.468
119.401
109.214
109.109
77.476
77.160
76.844
56.375
45.460
40.736
26.879
(thousandths)
X : parts per Million : Carbon13

Compound **5**

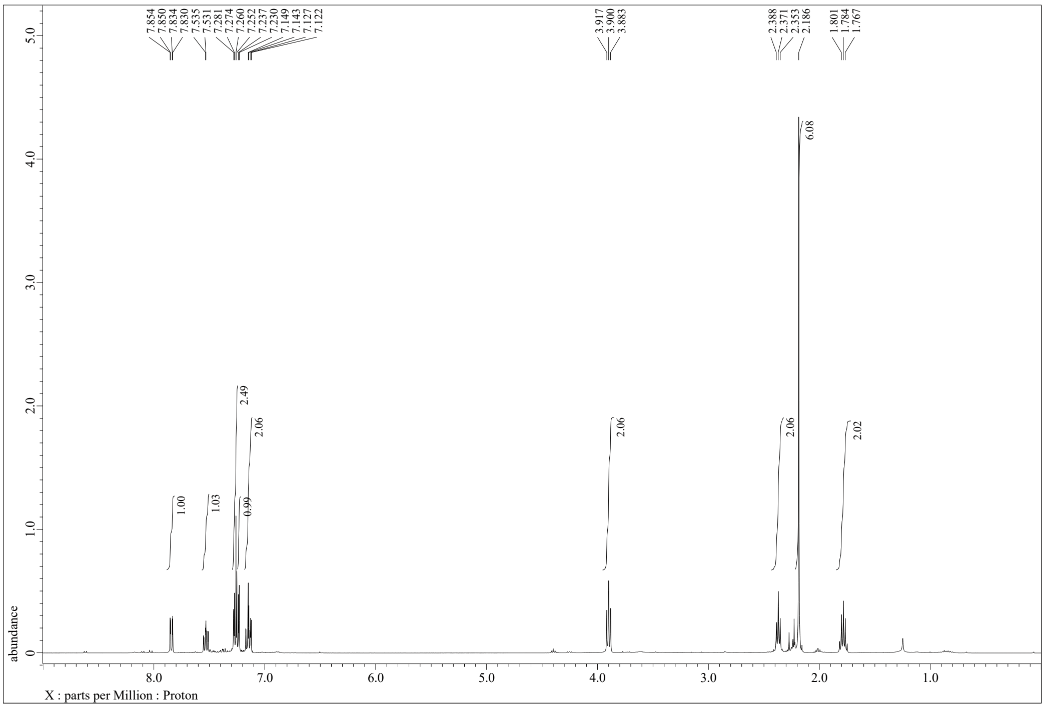

7.854
7.850
7.834
7.830
7.535
7.531
7.281
7.274
7.260
7.252
7.237
7.230
7.149
7.143
7.127
7.122
3.917
3.900
3.883
2.388
2.371
2.353
2.186
1.801
1.784
1.767
6.08
2.49
2.06
2.06
2.06
2.02
1.00
1.03
0.99
abundance
X : parts per Million : Proton


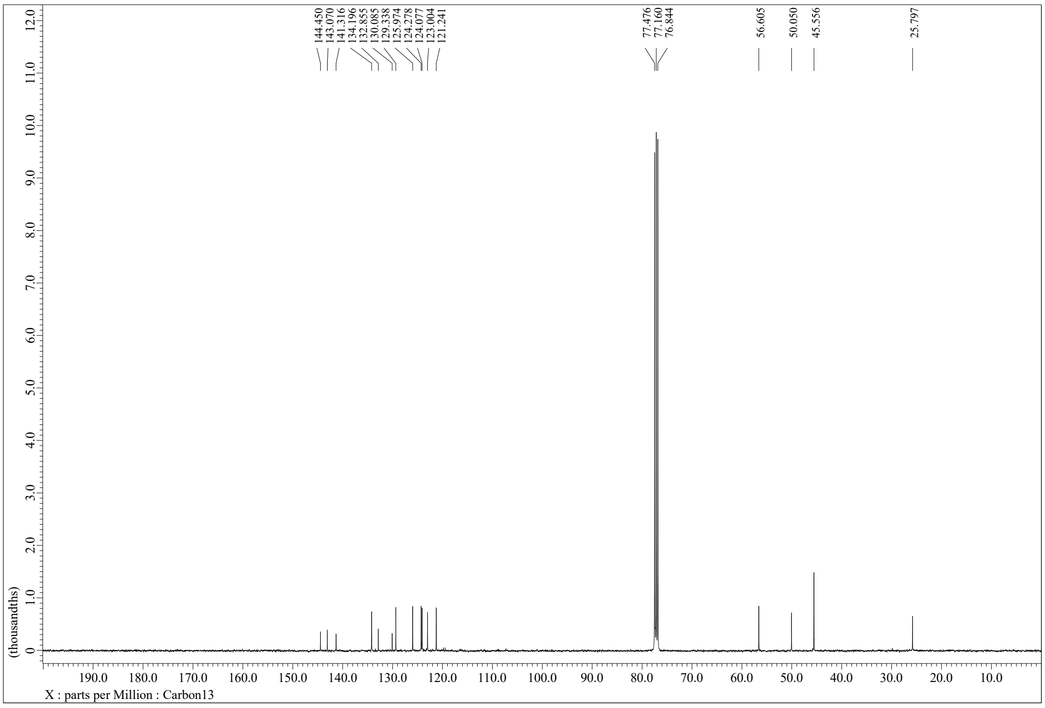

144.450
143.070
141.316
134.196
132.855
130.085
129.338
125.974
124.278
124.077
123.004
121.241
77.476
77.160
76.844
56.605
50.050
45.556
25.797
(thousandths)
X : parts per Million : Carbon13

## 4. Cartesian Coordinates (in Å) and Energies

**MCPZ**

B3LYP/6-31G(d,p)

E(RB3LYP) = –1414.559385

| | | | |
|---|---|---|---|
| C | 2.865802 | -1.383439 | -0.315847 |
| C | 1.614642 | -1.960341 | -0.09104 |
| C | 0.512866 | -1.17796 | 0.247533 |
| C | 0.631185 | 0.224932 | 0.328067 |
| C | 1.879859 | 0.808758 | 0.066893 |
| C | -1.760754 | 0.660476 | 0.1442 |
| C | -2.133526 | -0.69452 | 0.049026 |
| C | -3.376651 | -1.055814 | -0.468183 |
| H | -3.631221 | -2.108511 | -0.544512 |
| C | -4.28912 | -0.07621 | -0.863511 |
| C | -3.931986 | 1.266309 | -0.76898 |
| C | -2.673983 | 1.63357 | -0.287746 |
| H | 3.727945 | -1.994125 | -0.554975 |
| H | 1.497931 | -3.037255 | -0.161242 |
| H | 2.008237 | 1.882591 | 0.086763 |
| H | -5.263108 | -0.364069 | -1.245805 |
| H | -4.625103 | 2.040486 | -1.083815 |
| H | -2.406698 | 2.683063 | -0.25391 |
| N | -0.492035 | 1.006268 | 0.670921 |
| C | -0.272648 | 2.382456 | 1.089935 |
| H | -0.078807 | 3.073575 | 0.255655 |
| H | -1.153932 | 2.732162 | 1.630756 |
| H | 0.579401 | 2.415004 | 1.771806 |
| S | -1.03319 | -1.94624 | 0.682341 |
| C | 2.975809 | -0.000573 | -0.229832 |
| Cl | 4.533627 | 0.765129 | -0.524572 |
